# Do We Perceive Others Better than Ourselves? A Perceptual Benefit for Noise-Vocoded Speech Produced by an Average Speaker

**DOI:** 10.1371/journal.pone.0129731

**Published:** 2015-07-02

**Authors:** William L. Schuerman, Antje Meyer, James M. McQueen

**Affiliations:** 1 Max Planck Institute for Psycholinguistics, Nijmegen, the Netherlands; 2 Donders Institute for Brain, Cognition and Behaviour, Radboud University, Nijmegen, the Netherlands; 3 Behavioural Science Institute, Radboud University, Nijmegen, the Netherlands; The University of Chicago, UNITED STATES

## Abstract

In different tasks involving action perception, performance has been found to be facilitated when the presented stimuli were produced by the participants themselves rather than by another participant. These results suggest that the same mental representations are accessed during both production and perception. However, with regard to spoken word perception, evidence also suggests that listeners’ representations for speech reflect the input from their surrounding linguistic community rather than their own idiosyncratic productions. Furthermore, speech perception is heavily influenced by indexical cues that may lead listeners to frame their interpretations of incoming speech signals with regard to speaker identity. In order to determine whether word recognition evinces similar self-advantages as found in action perception, it was necessary to eliminate indexical cues from the speech signal. We therefore asked participants to identify noise-vocoded versions of Dutch words that were based on either their own recordings or those of a statistically average speaker. The majority of participants were more accurate for the average speaker than for themselves, even after taking into account differences in intelligibility. These results suggest that the speech representations accessed during perception of noise-vocoded speech are more reflective of the input of the speech community, and hence that speech perception is not necessarily based on representations of one’s own speech.

## Introduction

Speech production does not operate in a vacuum, free from the influences of its perceptual counterpart; the two processes are coupled and closely linked. [1]

To someone unfamiliar with the field of speech research, it may seem odd that such a statement needs to be expressed explicitly considering how natural the link between the production and perception of speech appears to be. Given the many dissociations found between speech production and perception, however, it may seem quite reasonable to treat production and perception as separate objects of study. For example, lesions to particular areas of the brain appear to solely hinder production abilities while leaving perceptual abilities intact (and vice-versa [2]), and it has been well-documented that children are able to perceive certain sounds that they are not yet able to produce [3]. Theories have indeed been developed for either production alone [4] or perception alone [5]. But at the opposite extreme, others propose that speech perception crucially relies on production mechanisms [6], in concordance with certain theories of action perception [7]. In this study, we test listeners’ perception of a type of spectrally manipulated speech, noise-vocoded speech, in which indexical cues have been largely eliminated. Testing perception of such speech allows us to investigate whether listeners are better at perceiving their own speech or the speech of another person in the absence of primary phonetic cues to speaker identity, and how this may enrich our understanding of the coupling between production and perception mechanisms.

Models of speech perception that argue that the production system plays a critical role, such as the motor theory of speech perception [8] and analysis by synthesis [9], have existed for some time and have undergone many revisions and changes [10]. Such theories have recently experienced a resurgence in interest due in great part to the growing body of neurobiological evidence demonstrating that speech motor areas (ostensibly only supposed to be activated during production) may become activated not only during production but also during perception [11]. Much of this neurobiological evidence has been gathered from experiments involving transcranial magnetic stimulation (TMS), in which targeted magnetic pulses are used to stimulate or inhibit activity in select areas of the brain [12]. By pairing TMS pulses with electromyography, which measures the activation of muscles via electrodes placed on the surface of the skin, it becomes possible to measure the degree of motor activation during the production or perception of an action. For example, muscle activation in lip regions involved in articulation has been found to be greater when listening to speech vs. non-speech (though only in response to left-hemisphere stimulation) [13]. More recent studies suggest that motor involvement may not always be necessary in order to perceive speech, but instead that motor areas may be recruited only during the perception of noisy, ambiguous, or non-native speech [14]. However, as proponents of motor involvement in speech perception have argued, pristine speech such as that found in laboratory conditions may be more likely to be the exception rather than the norm [15].

While neurobiological evidence suggests that motor areas may become activated during speech perception, it remains unclear what the functional role of this activation may be. One possibility is that this activation indicates parity between the mental representations accessed during production and perception. According to this view, perceiving an action requires accessing the same mental representations utilized when producing that action. This is in effect an argument for the common coding theory of perception, which argues that “percept codes and act codes are formed in the same format and are stored or maintained in a common representational medium” [7, p. 171]. One of the predictions of this theory is that perception is facilitated according to the degree to which the incoming sensory input matches the internal “action” representation; the more the action that we are perceiving matches with how we ourselves would produce it, the more easily the corresponding representation is activated.

Evidence in support of common coding has been provided by studies that have found perceptual facilitation for self-generated actions. For example, in a study by Knoblich, Seigerschmidt, Flach, and Prinz [16], participants were asked to write either the number “2,” or the first stroke in the number “2” without the horizontal line (a hook), utilizing a stylus and tablet. When later presented with recordings of only the hook stroke and asked to predict whether a horizontal stroke would follow (i.e. whether this was an instance of drawing the number “2” or simply drawing the hook part), participants were found to perform above chance accuracy when the stimulus was drawn from their own productions but only at chance when it had been produced by another participant. Similar perceptual advantages for self-generated stimuli have also been found for predicting where a thrown dart will land [17]. The authors argue that these results support a common coding account for action representations; our perception of some event, and the subsequent predictions that follow, are facilitated according to the degree of similarity between the actions that we observe and our own production of those actions.

Few studies have been undertaken to determine to what extent these effects may hold true in the realm of speech perception. If representations for speech sounds are shared for both production and perception, this predicts that speech perception ought to be facilitated for self-generated speech as well. A recent experiment on the perception of lip-read speech suggests that this may indeed be the case [18]. Two groups of ten participants were videotaped reading approximately 360 sentences aloud. These same participants were later tested on a subset of the 360 sentences, with an equal number of video clips drawn from every participant in the group (including each participant’s own recordings). Accuracy was scored as the percentage of words correctly identified. Fifteen of the 20 participants correctly identified a higher percentage of words when lip-reading themselves than when lip-reading other participants. This is rather startling, as speakers do not usually observe their own articulations and receive only auditory, tactile and proprioceptive feedback online. The results suggest that listeners do in fact utilize their production experience during perception, yet it is unclear to what extent this is restricted to the task of lip-reading. If auditory speech perception were also a form of action perception, this would predict that similar self-advantages ought to be found in auditory word recognition as well.

However, when comparing action perception to speech perception, it is crucial to recognize that speech perception is known to be influenced by indexical cues to speaker identity. For example, the perception of synthesized fricatives on a continuum between “sod” and “shod” can be influenced by the perceived gender of the speaker [19]. In a subsequent experiment, in which listeners were presented with audio stimuli that had been generated from the recordings of a speaker whose voice was judged to be non-prototypical for their gender were paired with video of either a male or female speaker, it was found that the visual cue to speaker gender further modulated the perceived fricative boundary. This demonstrates that auditory cues to gender and audio-visual integration of perceived gender can influence the perception of speech sounds. Later experiments replicated this audio-visual gender integration effect for vowel stimuli and further found that with audio-only presentation, the phoneme boundary could be modulated simply by instructing the listener to imagine the speaker as either male or female [20]. Shifts in phoneme categorization have also been found when participants associate a speaker with a specific social group (e.g. nationality) on the basis of an accompanying label [21]. These studies demonstrate that speech perception can be influenced by the perceived identity of the speaker as indexed by both linguistic and extra-linguistic cues. It is therefore not unreasonable to assume that recognizing stimuli as self-produced may also influence perceptual processing. This means that any benefit in recognizing one’s own speech could be the result not only of overlap in representations, as predicted by the common coding account, but also because the listener’s own speech was recognized as such.

If, in contrast, representations accessed for perception are fundamentally distinct from those accessed for production, those representations may be more reflective of the statistical properties of the speech in a given linguistic community. It is evident from experiments on cross-linguistic speech perception that perceptual identification of a speech sound depends on the sound’s distribution in the perceiver’s own language. For example, when tested on synthesized speech samples along a continuum between /l/ and /r/, American English speakers show categorical discrimination effects which reflect the bimodal distribution of these speech sounds in American English, whereas inexperienced Japanese speakers do not show a categorical discrimination effect [22]. Experienced Japanese listeners, however, who have been exposed to tokens of /l/ and /r/, and thus a bimodal distribution of sounds, perform similarly to the native English speakers. Studies differ with regard to whether perceptual effects are coupled to production ability; Bradlow, Akahane-Yamada, Pisoni, and Tohkura [23] found that Japanese speakers given intense perceptual training on the English /l/ ∼ /r/ distinction improved both their perception and production of the contrast, even up to three months following training. This suggests that perceptual skills may be tightly coupled to production skills. In contrast, Sheldon and Strange [24] found that participants were often better at producing a given phoneme (as judged by native English speakers) than they were at perceiving the same phoneme produced by themselves or other Japanese learners. At first glance, this would seem to suggest dissociation between production and perception. However, this study also found that four of the five Japanese participants made fewer errors when identifying /l/ and /r/ recordings produced by themselves compared to stimuli produced by other Japanese speakers or native English speakers. Further research by Borden, Gerber and Milsark [25] on Korean speakers suggests that individuals may differ with respect to whether perception is more accurate than production or vice-versa, though this may be related to length of time spent in an English-speaking environment [26]. These cross-linguistic perception studies thus provide conflicting evidence with regard to the common coding of speech representations.

At a broad level of granularity, speakers’ productions will almost necessarily reflect the statistical properties of their native language. It is therefore difficult to determine from cross-linguistic experiments whether decoding incoming speech in one’s native language utilizes representations that are more reflective of the listener’s own production idiosyncrasies or of the statistical properties of the speech community, as such fine-grained differences will most likely reside at the sub-phonemic level. In order to investigate which of these two alternatives may be the case, it is necessary to examine within a single language whether listeners show enhanced recognition when listening to themselves or to a “typical” speaker whose productions more closely approximate the average of their linguistic community.

## Experiment 1

In this study, we investigated whether spoken word recognition is facilitated more when words have been generated by the participants themselves or more when they were generated by a statistically average speaker. Experience listening to a single talker leads to an increase in intelligibility for subsequent stimuli from the same talker [27], even when this signal is extremely altered. For example, Remez et al. [28] examined the recognition of isolated sine-wave sentences. Accuracy was found to be greater when the test sentences and exposure sentences were produced by the same talker in the same modality rather than by different talkers. This suggests that even when the speech signal is distorted, listeners are still sensitive to talker-specific phonetic regularities and that exposure to these regularities can facilitate recognition of novel words produced by the same talker.

Due to the extensive experience speakers have with their own productions, we might expect speakers to be very sensitive to their own phonetic idiosyncrasies. This seems likely given that speakers have been shown to correct online for subtle (sub-phonemic) deviations from their intended speech targets [29]. Listening to one’s own voice via a recording is, however, a very different experience from monitoring one’s productions. A speaker hears their own voice via both air- and bone-conduction, resulting in a different psychoacoustic experience compared to when a speaker listens to recordings of their own voice. Yet certain phonetic properties, such as the length of a given stop burst or changes in amplitude, remain invariant between listening to one’s own voice while speaking and listening to recordings of one’s own voice. We therefore decided to utilize “noise-vocoded speech” (NVS) to examine the perception of self-produced speech. NVS preserves temporal and amplitude cues while eliminating fine-grained spectral detail [30]. This includes many of the primary cues, such as formant dispersion and pitch, that people use to distinguish voices from one another [31]. Many recent studies utilize NVS to manipulate the intelligibility of linguistic stimuli systematically. These studies have found that, even with relatively high levels of degradation, participants are quickly able to adapt to the spectral manipulation and accurately recognize the original utterance [32–34]. Importantly, several studies have found that talker-identification in NVS is greatly impaired [35–38]. This rather extreme manipulation therefore provides another benefit, in that it eliminates many linguistic cues to speaker identity that influence speech perception [19, 20]. It thus allowed us to ask whether there was a self-advantage in speech perception independent of listeners’ ability to recognize the speech as their own.

In Experiment 1, we examined listener accuracy for identifying single NVS words that had been produced either by the participants themselves or by a statistically average speaker. The experiment consisted of two sessions. First, in the recording session, native Dutch participants produced 120 Dutch words. After comparing the phonetic properties of the recordings, we selected from amongst the participants one speaker who differed least from all other participants in the sample. This model speaker acted as a proxy for a statistically average speaker of the linguistic community. The recordings were then converted into NVS stimuli. After a one-week interval, the same participants were presented with these NVS stimuli and asked to identify the original words. Participants were unaware that half of the stimuli were based on their own recordings and that the other half were based on the recordings of the selected average speaker.

If identical representations for spoken words are utilized for both production and perception, as common coding posits, then we would expect to find greater accuracy for self-produced NVS stimuli. Furthermore, if listeners’ access to these representations does not depend on the speaker’s identity, we would expect to find advantages for self-produced stimuli regardless of perceived speaker identity. However, if representations are more representative of the overall input of the linguistic community, then we predict that participants would be more accurate at identifying words generated by a speaker whose productions more closely align with this average.

### Materials and Methods

#### Participants

Twenty-eight female native speakers of Dutch between the ages of 19 and 26 (*μ* = 21.63), all with healthy vision and hearing, participated in the experiment. In order to minimize large between-speaker differences, only female participants were invited to participate. All 28 participated in the production task. One participant was selected to be the “average” model speaker, leaving 27 participants for the identification task, which was performed approximately one week after the production task.

#### Ethics Declaration

Ethical approval for this study was obtained from the Ethics Committee of the Social Sciences Faculty of Radboud University. Written consent was obtained from each participant on the first day of the study. Participants were informed that their participation was voluntary and that they were free to withdraw from the study at any time without any negative repercussions and without needing to specify any reason for withdrawal. All participants were reimbursed for their participation.

#### Stimuli

The stimulus set of 120 Dutch words was based on English materials developed for speech-in-noise recognition experiments by Bradlow and Pisoni [39]. 35 phonemic segments were represented in this set of words, though with different frequencies (e.g., /z/ appears in 10 words, while /b/ appears in only 6 words). Words in the stimulus set were divided into two groups according to frequency, phonological neighborhood density, and the average frequency of their phonological neighbors. Phonological neighbors were defined as words that differed from the target word by the deletion, addition, or substitution of a single phoneme. “Easy” words were those that had high frequency (*μ* = 226.53 per million), few phonological neighbors (*μ* = 9.2), and low neighborhood frequency (average frequency of all phonological neighbors; *μ* = 46.49 per million). “Hard” words, on the other hand, had relatively low frequency (*μ* = 16.71), larger phonological neighborhoods (*μ* = 20.85), and higher neighborhood frequency (*μ* = 470.98 per million). Thus, Easy words were likely to stand out as there were no or relatively few similar sounding words, while Hard words were more likely to be confused with more frequent, similar sounding words. Pilot tests confirmed that participants correctly recognized noise-vocoded versions of the Easy words more often than Hard words, validating the use of these parameters to distinguish the two conditions. All words utilized in this experiment were classified as 100% familiar according to a 559-participant age-of-acquisition study [40].

#### Production Task

During the recording session, participants were comfortably seated in a sound-attenuated booth. A pop-filter shielded microphone was placed approximately 10 cm away from each participant’s mouth. Participants were presented visually with the 120 stimulus words on a computer screen and asked to read them out loud in a “normal manner” within a specific recording window that was signaled visually. A black screen was presented for 500 ms, after which the target word appeared on the screen above a white box. After 250 ms a red circle appeared within the white box to mark the beginning of the recording and signal the participant to read the word out loud. Recording continued for 1750 ms, after which the red circle disappeared. At the end of each such trial, the participant was given the option to repeat the trial (e.g. if the participant had coughed during the recording) or to continue on to the next trial. The order of stimulus presentation was fully randomized for each participant. All productions were recorded using Presentation software (Version 0.70, www.neurobs.com).

#### Model Speaker Selection

Each recorded sound file was segmented and analyzed utilizing Praat [41]. Measurements taken from the participant recordings consisted of average word duration per speaker, average segment duration per speaker, duration of each word (120 variables), segment duration by word (e.g. /z/ in zuil’; 426 variables), and average segment amplitude (averaged across words; 35 variables). In addition, segments were also analyzed with respect to the NVS manipulation, which divided the sound file into six frequency bands. Therefore we included measurements of average amplitude by segment by frequency band (e.g., the average amplitude of each frequency band for all words in which the given segment appears; 210 variables) and standard deviation by segment by frequency band (210 variables). In order to find the most “average” speaker, participants were compared according to the aforementioned variables. Due to the fact that many of these variables were highly correlated, standard principal component analysis was used to reduce the number of dimensions for comparison from 1003 to 27. Euclidean distances between participants were calculated based on these 27 components, and the participant with the smallest average distance from all other participants was chosen as the average speaker.

#### Stimulus Preparation

The intensity of each of the total 3360 sound files (120 words * 28 participants) was normalized by root-mean-squared amplitude. The normalized sound files were then transformed into 6-band noise-vocoded speech [30]. These six spectral frequency bands corresponded to equally spaced distances along the basilar membrane [42]. Pre-experiment pilots determined that this level of degradation avoided ceiling and floor performance in the identification task.

#### Identification Task: Design

For each participant, each of the 120 target words was assigned to only one of two “Talker” conditions, a “Self” condition or an “Average Speaker” condition. The number of words from the Easy and Hard word lists was balanced across these Talker conditions (30 each). This ensured that each Talker condition included a range of words with equivalent and variable difficulty. Repetition of words across Talker conditions was avoided due to the possible confounding effect of acclimatization to the noise-vocoding manipulation. Order of stimulus presentation was randomized within the two Talker blocks. Order of block presentation was counterbalanced across participants.

#### Identification Task: Procedure

In the identification phase, participants attempted to identify noise-vocoded versions of the recorded words. A trial began with visual presentation of a fixation cross located in the center of a computer screen. The auditory stimulus was presented after 250 ms of silence. Following stimulus presentation, participants attempted to identify the target word by typing in their response via the computer keyboard. Once the participant confirmed their response by pressing the Enter key, the next trial began.

A 10-word practice session preceded the experimental blocks in order to familiarize participants with the noise-vocoded stimuli and the task. Feedback was given after each practice item, indicating whether or not the participant had correctly guessed the word. None of the words in the practice session appeared in the experimental blocks. Participants received no feedback during test blocks.

### Results

**Fig 1:**
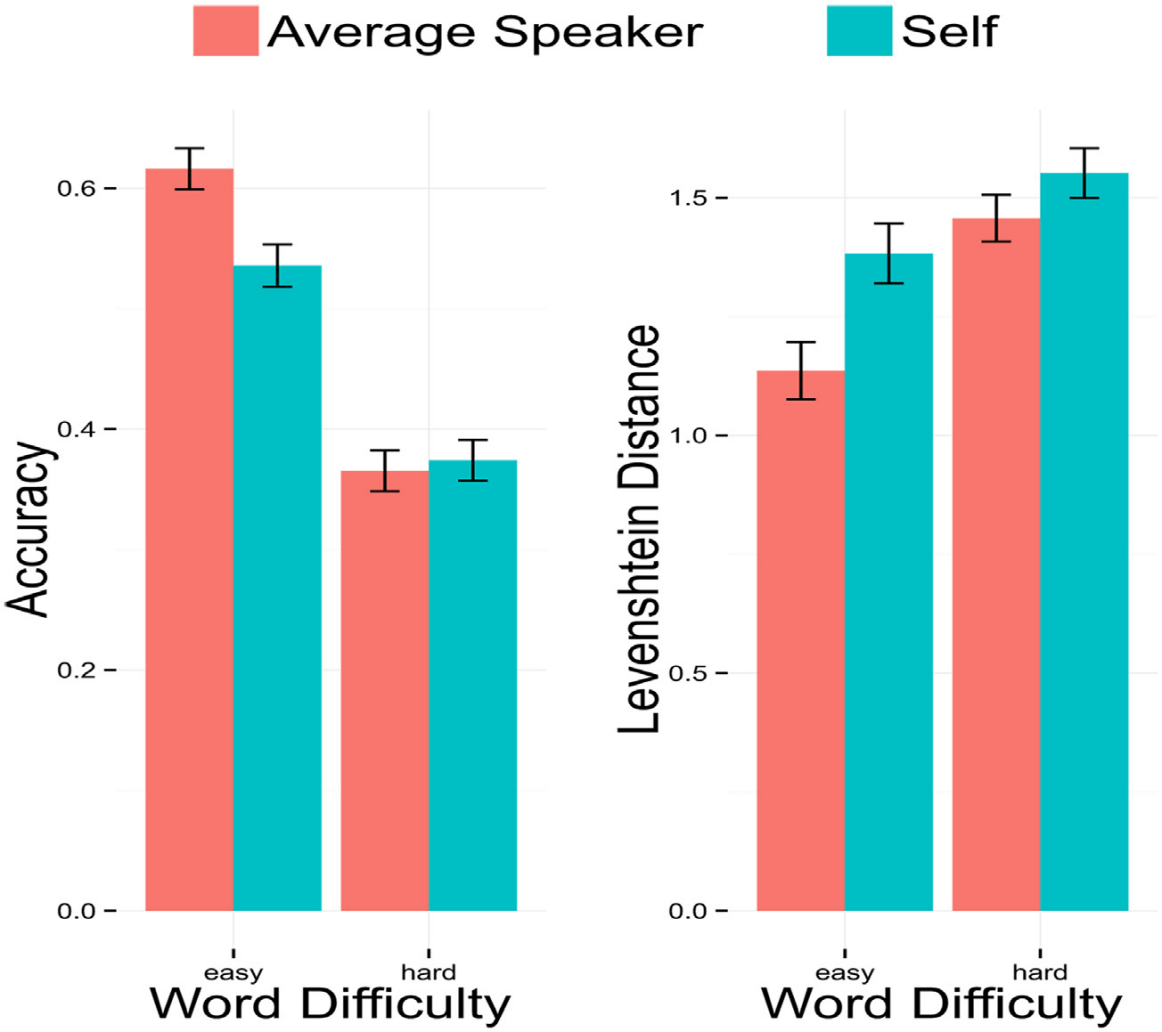
Average proportion of correct answers (Accuracy) and Levenshtein Distance for each word list, according to Talker, with standard error bars. For Levenshtein Distance, higher scores indicate greater inaccuracy.

Participants responded using standard Dutch orthography; given that Dutch orthography may represent the same sound in multiple ways (e.g., word final [t] can be spelled as either “t” or “d”), target words and participant responses were transcribed into a standardized broad phonemic script. Correspondence between target and response was measured utilizing these phonemic transcriptions rather than the raw orthographic input. The average percentages of correct responses (defined as 100% match between target and response transcription) by Talker and Word Difficulty are displayed in Fig 1. While individuals varied with respect to their accuracy, responses were much more accurate for Easy words (*μ* = 0.57, SE = 0.012) than Hard words (*μ* = 0.37, SE = 0.012) and moderately more accurate for words in the Average Speaker condition (*μ* = 0.49, SE = 0.012) than in the Self condition (*μ* = 0.45, SE = 0.012). For Easy words, participants were more accurate in the Average Speaker condition (*μ* = 0.62, SE = 0.017) than in the Self condition (*μ* = 0.53, SE = 0.017). For Hard words, accuracy was slightly greater in the Self condition (*μ* = 0.37, SE = 0.017) than in the Average Speaker condition (*μ* = 0.36, SE = 0.017).

Scoring responses in a binary format as either correct or false depending on a 100% match between target and response transcription fails to capture the detail contained within the transcribed responses. If the target word was “vacht” (fur) and a participant reported to have heard “macht” (power), then the participant did in fact accurately identify a substantial portion of the target word. For this reason, Levenshtein Distances [43] were computed between the target and response transcriptions. Levenshtein Distance is a metric that measures the number of edits (substitutions, additions, deletions) required to transform one string of characters into another. This method has been utilized, for example, to measure the linguistic distance between dialects of a given language [44]. According to this metric, a perfectly correct response is scored as 0 (no edits), while larger scores indicate greater inaccuracy.

**Fig 2:**
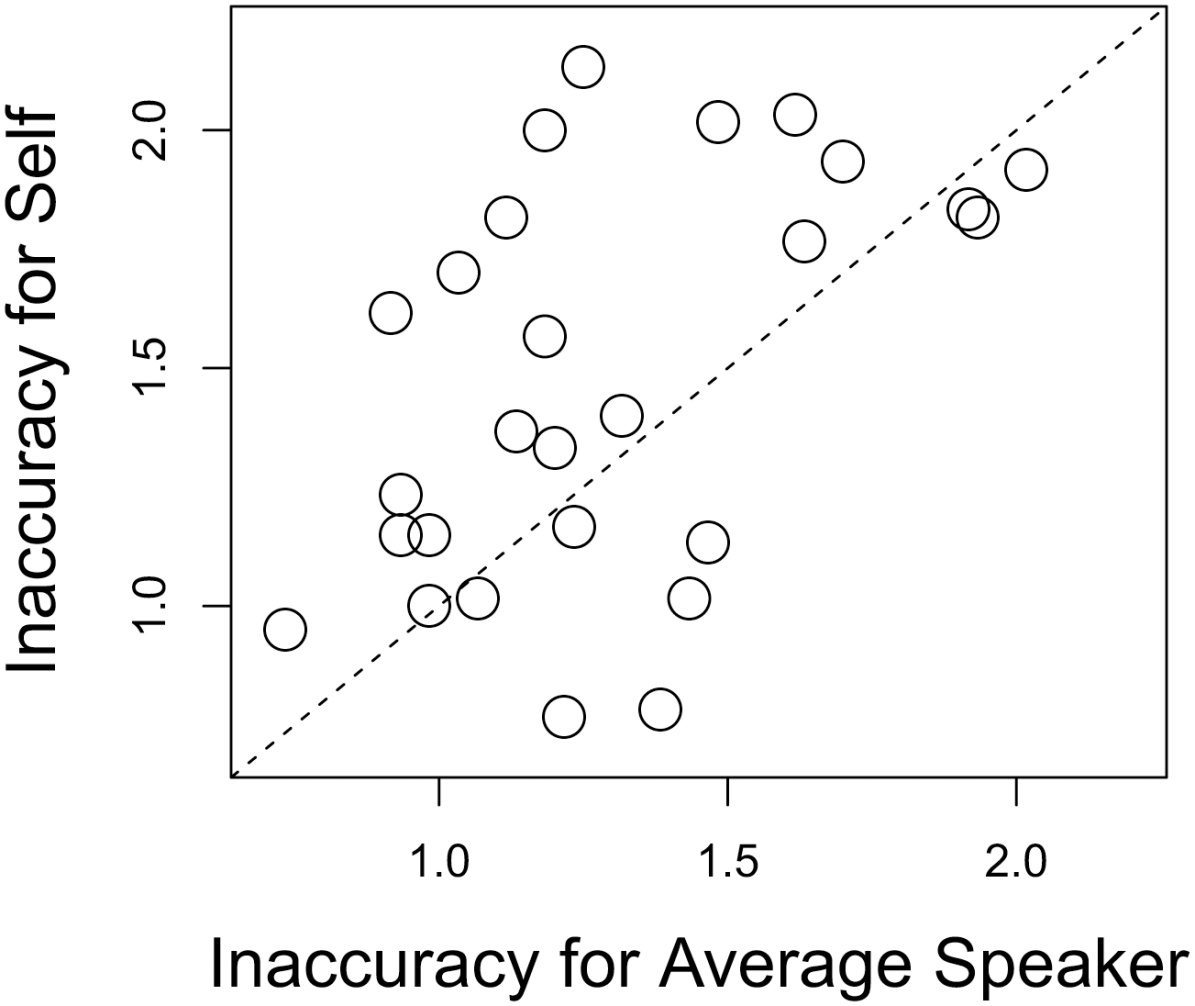
Average Levenshtein Distance for stimuli produced by the participant (Self) and by the Average Speaker. Higher scores indicate greater inaccuracy.

Average Levenshtein Distance measurements corresponded to the binary scored accuracy measurements; participants show greater inaccuracy (higher average Levenshtein Distance) for Hard words ((*μ* = 1.50, SE = 0.03) than for Easy words (*μ* = 1.26, SE = 0.04), as well as greater inaccuracy for words in the Self condition (*μ* = 1.47, SE = 0.04) than words produced by the Average Speaker (*μ* = 1.30, SE = 0.04). As Fig 1 shows, for Easy words, average Levenshtein Distance was greater in the Self condition (*μ* = 1.38, SE = 0.06) than in the Average Speaker condition (*μ* = 1.16, SE = 0.06); for Hard words, average Levenshtein Distance was also greater in the Self condition (*μ* = 1.55, SE = 0.05) than in the Average Speaker condition (*μ* = 1.46, SE = 0.05). An examination of each individual’s scores reveals that while some participants showed no difference in inaccuracy between Talker conditions and certain participants were more accurate for Self-produced stimuli, most participants were more accurate for the Average Speaker’s sound files than their own (Fig 2).

Due to the high number of zero scores (100% accurate responses) present in the data, we utilized a hurdle model to analyze the Levenshtein distance findings. A hurdle model is a combination of two models; it combines a binomial regression model to analyze zero vs. non-zero responses, as well as a zero-truncated Poisson model to analyze all responses greater than zero (i.e. those with at least one error). Given that the words varied between Talker conditions and were produced by different speakers, we decided to analyze the results using mixed-effects regression. All analyses were conducted in R using the glmmADMB package [45].

All statistical models included the maximal random effects structure justified by the experimental design [46]. This consisted of random intercepts for Item and Participant, random slopes for Talker (Self/Average Speaker) by Word, as well as random slopes for Talker, Word Difficulty, and the interaction between Word Difficulty and Talker by Participant.

We report the predictors entered into the binomial (*Binom*) and truncated Poisson (*TruncPoiss*) model at each step. The maximal fixed-effect structure was determined by likelihood comparison. Model comparison began with null models containing only the random effect structure. Next, we added a fixed effect of Word Difficulty type, which was found to substantially improve the fit of both models (*Binom*: AIC = 3767.66, BIC = 3816.33, LogLik = -1875.8, *χ* ^2^ = 19.18, p < 0.001; *TruncPoiss*: AIC = 5223.34, BIC = 5266.88, LogLik = -2603.7, *χ* ^2^ = 14.34, p < 0.001). Addition of a fixed effect of Talker also significantly improved both models (*Binom*: AIC = 3764.62, BIC = 3819.37, LogLik = -1873.3, *χ* ^2^ = 5.04, p < 0.05; *TruncPoiss*: AIC = 5221.02, BIC = 5270.01, LogLik = -2601.5, *χ* ^2^ = 4.32, p < 0.05). Inclusion of an interaction term between Talker and Word Difficulty did not significantly improve either model.

Parameters for the fixed effects are reported in Table 1 for the final versions of the binomial and truncated Poisson model, both with reference categories of “Easy” (Word Difficulty) and “Average Speaker” (Talker). For the binomial model, the fixed effect of Word Difficulty reveals that when comparing zero (correct) and non-zero (incorrect) responses, the likelihood of a non-zero response was significantly greater for more difficult words. However, for the truncated Poisson model, the negative slope indicates that Levenshtein Distance decreased in the Hard condition, possibly because of the large number of trials in this condition in which there was only one error between target and response transcription.

**Table 1:** Results of the binomial and truncated Poisson mixed-effects regression analyses.

	Binomial				Truncated Poisson			
Fixed Effects:	Estimate	Std. Error	Z-value	Pr(> ∣*z*∣)	Estimate	Std. Error	Z-value	Pr(> ∣*z*∣)
(Intercept)	-0.530	0.194	-2.73	0.0063**	0.874	0.066	13.24	<2e 16***
Word Difficulty (Hard)	1.15	0.253	4.55	<5.3e-06***	-0.312	0.080	-3.79	0.0001***
Talker (Self)	0.264	0.114	2.32	0.0203*	0.075	0.036	2.08	0.0379*

Fixed effects reported in reference to Talker (Average Speaker) and Word Difficulty (Easy).

* *p* <.05,

** *p* <.01,

*** *p* <.001.

### Discussion

The results suggest that participants were more accurate at identifying noise-vocoded words produced by an average speaker than by themselves. Differences between Talker conditions were greater for Easy words than Hard words. This is somewhat surprising because factors that would improve intelligibility would be expected to have a stronger effect when stimuli are more difficult to decode. For example, in a study on speech recognition in noise, Bradlow and Pisoni [39] found greater differences between easy and hard words at a fast speech rate than at a slow speech rate. In the same study, however, the authors also compared speakers’ perception of easy and hard words in single and multi-talker conditions; they found that the differences in accuracy between easy and hard words was greater in the single-talker condition than in the multi-talker condition. The authors argue that the increase in accuracy in the single talker condition stems from listeners’ “ability to take advantage of consistent surface information about a particular talker’s voice” [p. 11]. These results mirror our own; we find greater differences between Easy and Hard words in the Average Speaker condition compared to the Self condition. This may suggest that the typicality of the speaker (i.e. how closely the speaker’s productions align to the statistical average of the community) may impart an advantage similar to that provided by repeated exposure to a single talker’s voice.

Due to the fact that each participant listened to a different set of sound files (Self vs. Average Speaker), differences in intelligibility across talkers may have led to the observed pattern of results. For example, with respect to the observed individual variation (Fig 2), it may have been the case that the participants who showed greater accuracy for their own sound files than for those produced by the Average Speaker had simply enunciated more clearly during the recording session; conversely, those participants who were more accurate in the Average Speaker condition than in the Self condition may have enunciated less clearly or their recordings had been rendered more unintelligible by the noise-vocoding procedure. If so, we would expect to find no effect of Talker after having accounted for the general intelligibility of each speaker.

In order to assess this possibility, we conducted a control experiment to determine the average intelligibility of each speaker. Noise-vocoded stimuli from each talker in the main experiment were presented to new participants, who performed the same open-response identification task. The percentage of correctly identified words constituted a given talker’s “intelligibility score.” We predicted that intelligibility scores from this control experiment would correlate with a participants’ average accuracy in the Self-condition in Experiment 1. The main purpose of Experiment 2, however, was to establish whether the advantage for the Average Speaker’s words in Experiment 1 would remain after talker intelligibility was taken into account.

## Experiment 2: Determining Speaker Intelligibility

### Materials and Methods

#### Participants

Sixteen female native speakers of Dutch, between the ages of 19 and 28 (*μ* = 22.63), all with reported healthy vision and hearing, took part in this control experiment. To maximize similarity to Experiment 1, only female participants were invited. None of them had participated in Experiment 1. All were paid for taking part in the experiment.

#### Stimuli & Design

A set of 112 noise-vocoded words were selected from the original set of experimental materials. In order to account for the variability in word difficulty, these 112 words were divided into four groups based on average by-word accuracy in Experiment 1 (high accuracy, mid-high accuracy, mid-low accuracy, and low accuracy). Each control participant was presented with four stimuli from each talker (one from each word difficulty group), randomly selected from the recordings obtained from the 28 talkers from Experiment 1. This resulted in a total of 64 words per talker (16 per word difficulty group), and ensured that any word level variations in intelligibility would not affect aggregated intelligibility scores. Stimuli were presented in two blocks, with each block repeated twice (ABAB). Order of presentation was pseudo-randomized such that no Talker was repeated twice in a row.

#### Procedure

The identification task was the same as in Experiment 1.

### Results

In order to determine whether the participants differed greatly with respect to how intelligible they perceived each talker to be (defined by average Levenshtein Distance), t-tests were performed on the cumulative distribution of results after each new participant was added in order to determine whether this distribution changed significantly. For example, the average scores for participants one through three were compared to the scores for participants one through four. After four control participants, the *t*-statistic (df = 52.592) was less than 1, suggesting that already after only a few control participants, incorporating results obtained from a new control participant did not significantly alter the distribution of results. Comparing the distribution of all 16 participants to the distribution of the 15 previous participants resulted in a *t*-statistic of -0.33 (df = 54). The negligible difference between these two distributions allowed us to assume that with 16 participants we had achieved a reasonably reliable measure of the intelligibility of each participant that was unlikely to change by adding additional control participants. Testing was therefore terminated at this point. Average talker intelligibility given all 16 participants’ data (as measured by Levenshtein Distance) was 1.6 (sd = 0.35), with a minimum (most intelligible) of 0.78 and a maximum (least intelligible) of 2.16.

**Fig 3:**
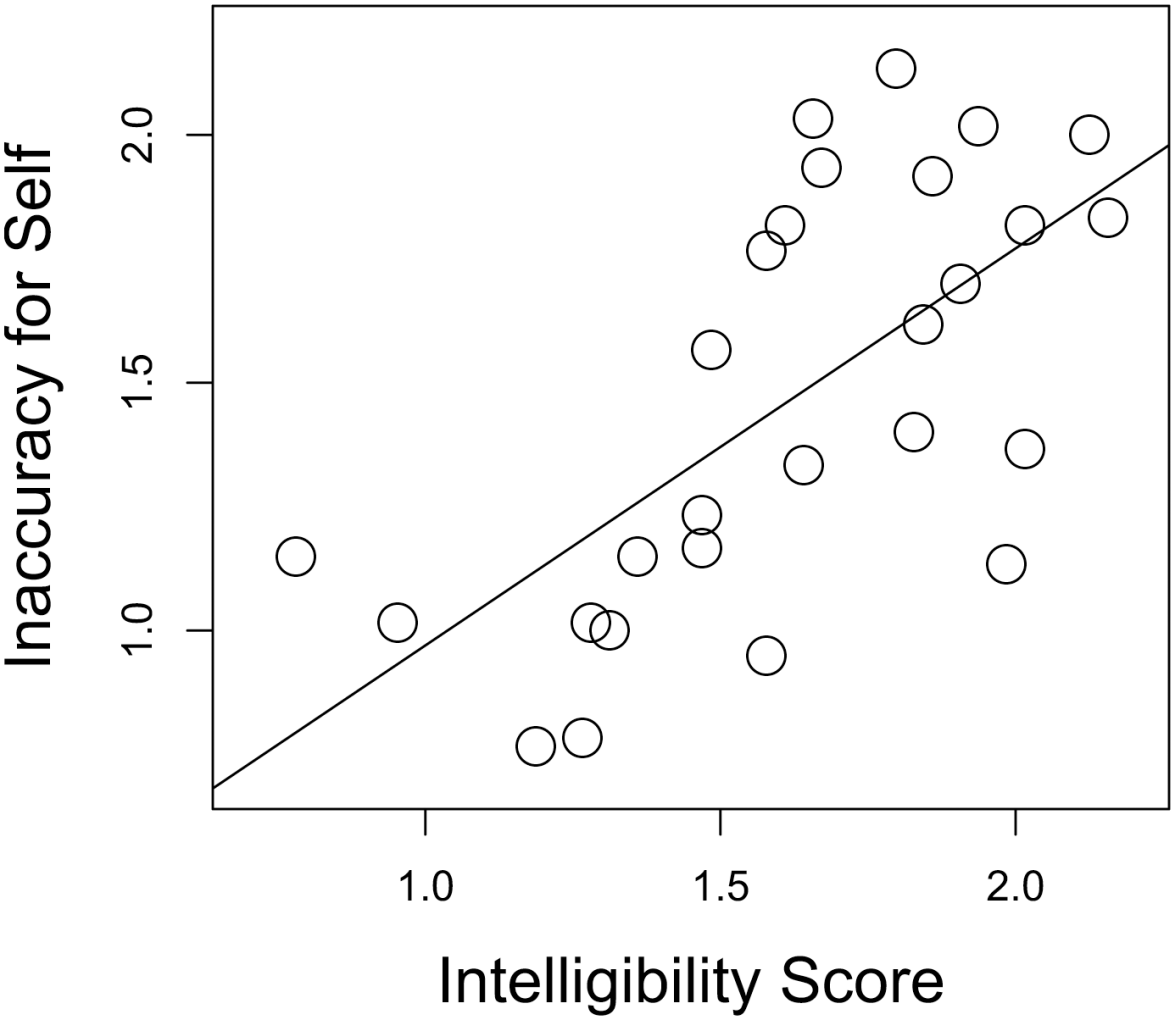
Intelligibility ratings by Levenshtein Distance for each speaker (Experiment 2) plotted against that speaker’s inaccuracy for Self-produced stimuli (Experiment 1), with regression line.

Accuracy in Experiment 1 in the Self-Condition is plotted against Intelligibility in Fig 3. As expected, one-tailed t-tests confirmed a significant correlation between accuracy for self-produced stimuli and the intelligibility ratings obtained in Experiment 2 (Pearson’s *r* = 0.66, *t*(25) = 4.388). The positive correlation reveals that if the control participants found a given talker’s speech to be more intelligible, then that talker was more likely to have recognized their own speech more accurately in Experiment 1.

The main purpose of obtaining intelligibility scores for each participant was to determine whether the effect of Talker found in Experiment 1 could be solely explained by differences in participant intelligibility. Therefore the data from Experiment 1 was reanalyzed with intelligibility scores included in the model. A fixed effect of Intelligibility was found to significantly improve the null binomial and null truncated Poisson models (*Binom*: AIC = 3777.66, BIC = 3826.33, LogLik = -1880.8, *χ* ^2^ = 9.18, p < 0.01; *TruncPoiss*: AIC = 5232.72, BIC = 5276.26, LogLik = -2608.4, *χ* ^2^ = 4.96, p < 0.05). Crucially, after adding fixed effects of Word Difficulty and Intelligibility, the fixed effect of Talker still significantly improved the fit of both models (*Binom*: AIC = 3756.58, BIC = 3817.4, LogLik = 1868.3, *χ* ^2^ = 6.00, p < 0.05; *TruncPoiss*: AIC = 5218.42, BIC = 5272.85, LogLik = -2599.2, *χ* ^2^ = 4.08, p < 0.05). The slope of the fixed effect for Talker remained relatively unchanged in both models (Table 2; *Binom*: *β* = 0.267, Z = 2.54, p < 0.05; *TruncPoiss*: *β* = 0.07, Z = 2.02, p < 0.05). No significant interaction between Talker and Word Difficulty was found. This analysis reveals that greater accuracy for identifying words produced by the model speaker compared to the participants’ own productions, as observed in Experiment 1, cannot be accounted for simply by differences in intelligibility between each participant’s noise-vocoded words and those of the model speaker.

**Table 2:** Results of the binomial and truncated Poisson mixed-effects regression analyses.

	Binomial				Truncated Poisson			
Fixed Effects:	Estimate	Std. Error	Z-value	Pr(> ∣*z*∣)	Estimate	Std. Error	Z-value	Pr(> ∣*z*∣)
(Intercept)	-1.722	0.396	-4.35	<1.42e 05**	0.579	0.148	3.93	<8.64e 05***
Intelligibility	0.734	0.213	3.45	0.00057***	0.181	0.080	2.25	0.02*
Word Difficulty (Hard)	1.156	0.254	4.56	<5.2e-06***	-0.310	0.080	-3.87	0.00011***
Talker (Self)	0.267	0.105	2.54	0.011*	0.073	0.036	2.02	0.04*

Intelligibility ratings are based on Levenshtein Distance, therefore greater values indicate that the speaker is less intelligible.

* *p* <.05,

** *p* <.01,

*** *p* <.001.

## General Discussion

In this study, we investigated whether the speech representations accessed during perception of noise-vocoded speech are more reflective of an individual’s own productions or of the statistical average of the individual’s speech community. Common coding theory [7] predicts that perception should be more accurate when the listener is presented with self-generated stimuli, as has been found in experiments on the perception of self vs. other-generated actions [16–18]. If, in contrast, speech representations accessed during perception of noise-vocoded speech are distinct from those used in production and reflect exposure to a range of speakers, listeners should be more accurate at identifying stimuli generated by a speaker whose productions more closely approximate the statistical average of their community. We therefore tested participants on the recognition of noise-vocoded words that had either been produced by themselves in an earlier recording session or by a “statistically average” speaker chosen from the participant population. The pattern of results suggests that most speakers were more accurate at identifying words produced by the statistically average speaker than their own recordings. The results of the control experiment suggest that this advantage for the statistically average speaker was still significant even after taking into account intelligibility differences between the participants’ noise-vocoded words.

Given that self-advantages have been found in the visual modality, especially for lip-reading [18], why is it that we do not observe these effects in auditory word recognition? Noise-vocoding was utilized as a means of systematically degrading the stimuli while preserving temporal and intensity-related idiosyncrasies. It may have been the case that this manipulation eliminated crucial phonetic cues that listeners make use of when both monitoring their own speech and when listening to others. However, this seems unlikely, since the noise-vocoding manipulation preserved talker-specific differences in temporal parameters that listeners are known to be sensitive to, such as voice-onset time [47]. Furthermore, if listeners were not sensitive to variations in these parameters, we would expect to find no difference in accuracy between the Talker conditions independent of general intelligibility.

In experiments on action perception using visual stimuli, participants were often aware of the identity of the producer [17, 18]. Therefore, it may be the case that self-advantages are dependent on the perceiver consciously recognizing the speech as self-produced. In addition to degrading the stimuli in order to increase the difficulty of word recognition, noise-vocoding was used in our study as a means of preventing possible influences on perception arising from perceived speaker identity [19–21]; noise-vocoding eliminates phonetic cues often used to identify speakers [31]. This appears to have been effective, because during debriefing only two participants guessed that they had at some time heard their own voices. When asked what led them to this realization, both participants reported that they had remembered pronouncing a certain word with precisely the same duration during the recording session. Neither of these participants, however, recognized that the study had presented one talker per block, and the majority of the participants did not notice that voices differed between blocks. In the experiments conducted by Knoblich, Seigerschmidt, Flach and Prinz [16], in which participants attempted to predict aspects of handwriting strokes produced by themselves and other participants, advantages for self-produced stimuli were found without explicit cues to author identity. Knobich and Flach [17] argue that self-advantages should not depend on the perceiver’s awareness of whether the stimuli were self-produced. Furthermore, they argue that when information about the source of an observed action is sparse, imagined actions are more easily integrated, causing self-other differences to emerge even more swiftly than when identity cues are present. Fundamentally, the common coding theory [7] predicts that self-advantages should not depend on awareness of the identity of the producer. If participants automatically compare incoming speech to their own production representations, we would expect to find an advantage for self-produced speech regardless of whether the listener is able to identify the voice. Our results suggest, in contrast, that in the absence of cues to speaker identity, listeners decode incoming productions with reference to the statistical average of their community.

The use of NVS stimuli was also likely to have eliminated any phonetic cues that listeners unconsciously use to frame incoming speech with respect to speaker identity. Therefore it remains possible that, were such cues present in the stimuli, listeners would have performed differently. For example, Remez et al. [28] found that when listeners were given exposure to sine-wave sentences, they performed better when later attempting to identify single sine-wave words produced by the same talker. However, participants who were given exposure to clear versions of the same sentences produced by the same speaker did not show any benefit of exposure; both speaker-specific and modality-specific experience were necessary in order to facilitate word recognition. Nevertheless, as mentioned above, common-coding suggests that the same representations are accessed during both production and perception regardless of the identity of the perceived speaker. Therefore, while it remains possible that listeners may be more accurate at recognizing self-produced speech when they are aware that stimuli are self-produced, the current results challenge the common-coding prediction that self-produced speech will necessarily be recognized more accurately than the speech of others.

It is also important to consider why we do find advantages for the speech produced by the model speaker. For example, it may be the case that listeners assumed that the noise-vocoded speech was produced by an average talker, and this framing lead to facilitation in perception for the stimuli produced by the model speaker. This interpretation would be consistent with “talker normalization” [48, 49], a theory which argues that listeners utilize information about the talker, and frame incoming speech with regard to the perceived identity of the talker, in order to overcome the considerable amount of uncertainty in speech and variability between speakers [50]. With regards to our experiment, assigning the identity of “average speaker” to the NVS stimuli would facilitate perception when the durational and amplitude properties of the stimuli fell closer to the statistical average of the community. However, given how abnormal NVS sounds (the stimuli were often described by our participants as sounding as if they were produced by a “demon” rather than by a normal person), it seems implausible that listeners identified the producer of this speech as an average speaker of their community.

We suggest instead that listeners may have either assumed that the NVS was not self-produced given how strange it sounded compared to the listeners’ own speech (or any other normal speaker) and thus must have been produced by some “other”, or failed to make any explicit assumptions (positive or negative) about the source of the NVS. Importantly, no matter what assumptions the listeners did or did not make, they appear to have adopted a “default” perceptual strategy, leading to facilitation in the identification of speech produced by the average speaker. This strategy may depend on the lack of indexical information in NVS. For example, listeners have been found to generate more fine-grained expectations about the phonological qualities of upcoming words when these words are embedded in carrier sentences pronounced with the listener’s own regional accent [51]. However, when presented with a carrier sentence in a non-native regional accent, listeners do not make such fine-grained predictions. This demonstrates that listeners are able to draw upon more fine-grained linguistic representations to facilitate speech perception, but may only do so in the presence of indexical information cueing them to access such representations. In the context of visual action perception, the “default strategy” may involve accessing representations that more closely align with the participant’s own production experience [16], leading to advantages for self-produced stimuli. Yet our results suggest that during speech perception, in the absence of vocal cues to identity, a video showing the participant’s own face [18], or other indexical cues, the listener adopts a decoding strategy that relies on more general representations, and specifically not representations aligning to the listener’s own productions.

This view complements talker normalization theory by suggesting that unless given evidence otherwise, the most efficient strategy during speech perception is to access representations that are more likely to match the input, that is, statistically average representations. However, given that linguistic and extra-linguistic cues to identity are almost always present to some extent during normal speech perception, it is important to consider how this may affect the interpretation of our findings. With regard to the implications for clear speech, it may be the case that when faced with unfamiliar speech, e.g. speech in an non-native language produced by an unfamiliar speaker, listeners access statistically average representations from their own linguistic community. Yet when additional information is available, indexical cues may “fine-tune” underlying representations [51], or cue the listener to utilize alternative processing strategies.

We hypothesized that if listeners utilize a “common-code” for both production and perception [7], they should be more accurate at recognizing words based on their own recordings rather than those based on a speaker whose productions are more representative of the statistical average of the participant population, and that such effects should be found even in the absence of cues to the identity of the talker. While some participants were more accurate for self-produced stimuli than stimuli produced by the average speaker, the results of this study show an overall advantage for the stimuli produced by the average speaker, suggesting that spoken word recognition does not necessarily rely on common representations for production and perception. One of the advantages of a common-coding approach is that it eliminates the need to posit separate representations for speech sounds or word forms for both production and perception. Yet in so doing, it complicates the question of how people perceive actions that differ from their own. As Hickock remarks, “…we are fully capable of understanding actions we have never produced…it would be surprising, maladaptive even, if all observed actions resulted in the activation of the same motor program in the observer” [52]. If comprehension critically relied on accessing the very same representations utilized in production, then we would expect that people who deviate greatly from the norm in their production would also suffer perceptually, yet this does not appear to be true [2]. While self-advantages may be found for certain actions during visual perception, when faced with the task of recognizing words that vary across a number of speakers, the listener develops representations that extracts relevant information from different speakers and thus facilitates perception when faced with a novel speaker [53].

## Conclusion

This study examined one facet of the debate on the nature of representations of speech, namely whether incoming speech is decoded with reference to representations that are more reflective of the overall speech community or more reflective of the listener’s own productions. Our results suggest that, in the absence of indexical cues leading the listener to frame incoming noise-vocoded speech with respect to the identity of the speaker, the representations accessed during speech perception more closely align to the statistical average of the linguistic community rather than the speaker’s own productions. This may suggest that indexical cues lead to fine-tuning of these underlying representations, but future research is needed to determine how and to what extent these results generalize to speech perception when such cues are present. The current results nevertheless already indicate that, contrary to a strict common-coding account, speech perception is not necessarily based on representations of one’s own speech.

In interpreting our results, we emphasize the demands placed upon a listener to decode incoming speech; given a population in which different talkers may vary greatly with respect to the pronunciation of the same words or speech sounds, it is likely to be more efficient to attempt to decode incoming speech utilizing representations that closely approximate the statistical distribution of the community rather than one’s own productions, which may deviate substantially from the average. While it is crucial to integrate research on speech production and perception, such research should take into account the differing demands on a speaker compared to a listener and how this may be reflected in the nature of the representations utilized for both types of language processing.

## Supporting Information

S1 Table Word lists organized by Word Difficulty. Word is given in Dutch orthography. The second column contains a modified CPSAMPA transcription, which was utilized to calculate Levenshtein Distances. In the original CPSAMPA transcriptions, certain consonant sounds were designated by combinations of characters. In the modified versions, each consonant is designated by a single character. The third column refers to the lexical frequency of the word (as measured by Subtlex per-million). PTAN refers to the Number of phonological neighbors. PTAF refers to the average frequency of a word’s phonological neighborhood. All information obtained from the Northwestern University DutchPOND database (http://clearpond.northwestern.edu/dutchpond.html).
(DOCX)
