# Supplementary material for: Do We Perceive Others Better than Ourselves? A Perceptual Benefit for Noise-Vocoded Speech Produced by an Average Speaker
Source: PLoS One. 2015 Jul 2;10(7):e0129731. doi: 10.1371/journal.pone.0129731 (PMC4489924; doi:10.1371/journal.pone.0129731)
Supplement: S1 Table — Word is given in Dutch orthography. The second column contains a modified CPSAMPA transcription, which was utilized to calculate Levenshtein Distances. In the original CPSAMPA transcriptions, certain consonant sounds were designated by combinations of characters. In the modified versions, each consonant is designated by a single character. The third column refers to the lexical frequency of the word (as measured by Subtlex per-million). PTAN refers to the Number of phonological neighbors. PTAF refers to the average frequency of a word’s phonological neighborhood. All information obtained from the Northwestern University DutchPOND database (http://clearpond.northwestern.edu/dutchpond.html). (DOCX) [file pone.0129731.s001.docx]

Appendix: Word Lists

PTAN: Number of phonological neighbors.

PTAF: Average frequency of phonological neighborhood.

Easy Word List:

| Word | Transcription | Translation | Freq_per_million | PTAN | PTAF |
| --- | --- | --- | --- | --- | --- |
| hotel | h.oU.t.E.l | hotel | 88.7274 | 1 | 6.6546 |
| onzin | O.n.z.I.n | nonsense | 111.2295 | 1 | 0.8918 |
| tafel | t.2.f.5.l | table | 83.3992 | 3 | 2.4545 |
| mens | m.E.n.s | man | 144.6623 | 7 | 26.9384 |
| schat | s.x.a.t | treasure | 264.0328 | 17 | 39.9018 |
| zelf | z.E.l.f | itself | 437.4629 | 3 | 138.892 |
| eind | Ei.n.t | end | 83.1705 | 7 | 2.95 |
| thuis | t.9y.s | home | 399.4793 | 11 | 86.9666 |
| agent | 2.G.E.n.t | agent | 186.5792 | 1 | 4.1162 |
| gezin | G.5.z.I.n | family | 79.8547 | 5 | 139.389 |
| wapen | 0.2.p.5.n | weapon | 140.5918 | 11 | 78.8069 |
| aarde | 2.r.d.5 | earth | 100.0699 | 6 | 45.8692 |
| geld | G.E.l.t | money | 793.6076 | 11 | 17.6021 |
| maand | m.2.n.t | month | 93.644 | 15 | 60.2554 |
| mama | m.2.m.2 | mama | 206.4514 | 1 | 1.6236 |
| plan | p.l.a.n | plan | 143.336 | 6 | 8.6364 |
| schip | s.x.I.p | ship | 115.2771 | 10 | 3.8464 |
| stem | s.t.E.m | voice | 86.5321 | 9 | 43.139 |
| papa | p.2.p.2 | papa | 223.2822 | 2 | 6.6317 |
| spel | s.p.E.l | game | 95.1076 | 10 | 88.3661 |
| stop | s.t.O.p | stop | 301.9706 | 16 | 24.0842 |
| auto | Vu.t.oU | car | 457.9983 | 2 | 91.8946 |
| kamer | k.2.m.5.r | room | 275.238 | 7 | 14.9654 |
| broer | b.r.u.r | brother | 245.6241 | 4 | 30.6373 |
| kaart | k.2.r.t | map | 79.6718 | 20 | 17.4368 |
| mond | m.O.n.t | mouth | 165.9295 | 20 | 50.5346 |
| fout | f.Vu.t | error | 165.2663 | 18 | 53.4728 |
| idee | i.d.eI | idea | 482.9929 | 0 |  |
| wagen | 0.2.G.5.n | car | 77.8195 | 16 | 76.739 |
| dame | d.2.m.5 | lady | 82.7361 | 3 | 56.6667 |
| foto | f.oU.t.oU | photo | 119.1646 | 2 | 0.5374 |
| plek | p.l.E.k | place | 177.4092 | 7 | 4.4625 |
| soort | s.O3.r.t | kind | 222.0244 | 14 | 43.2252 |
| begin | b.5.G.I.n | beginning | 193.1194 | 6 | 41.2994 |
| geval | G.5.v.a.l | case | 137.2074 | 5 | 28.562 |
| geest | G.eI.s.t | spirit | 94.9704 | 9 | 69.3914 |
| paard | p.2.r.t | horse | 83.6279 | 18 | 42.2472 |
| prijs | p.r.Ei.s | price | 86.6007 | 4 | 25.0403 |
| vraag | v.r.2.x | question | 436.6854 | 9 | 122.2365 |
| twee | t.0.eI | two | 1007.5139 | 5 | 13.0393 |
| grond | G.r.O.n.t | ground | 110.2461 | 5 | 40.3527 |
| hulp | h.Y.l.p | help | 239.7699 | 8 | 41.4652 |
| spijt | s.p.Ei.t | regret | 665.776 | 17 | 3.6642 |
| stap | s.t.a.p | step | 126.5281 | 17 | 53.3777 |
| trek | t.r.E.k | pull | 125.7277 | 10 | 13.2062 |
| rest | r.E.s.t | rest | 175.0309 | 18 | 43.2178 |
| rust | r.Y.s.t | rest | 75.3955 | 14 | 18.6341 |
| gang | G.a.N | corridor | 110.795 | 13 | 144.9526 |
| derde | d.E.r.d.5 | third | 76.4931 | 1 | 24.6287 |
| leger | l.eI.G.5.r | army | 107.9822 | 8 | 9.9561 |
| reden | r.eI.d.5.n | reason | 163.6656 | 16 | 34.2018 |
| kerk | k.E.r.k | church | 79.4888 | 7 | 102.1117 |
| stel | s.t.E.l | set | 214.9354 | 17 | 64.7202 |
| zorg | z.O.r.x | care | 218.8229 | 2 | 28.8935 |
| zaak | z.2.k | case | 239.3354 | 15 | 95.6107 |
| regel | r.eI.G.5.l | rule | 77.3164 | 6 | 21.3396 |
| kost | k.O.s.t | costs | 80.4035 | 17 | 98.6239 |
| nacht | n.a.x.t | night | 204.439 | 13 | 148.8489 |
| werk | 0.E.r.k | work | 680.2285 | 8 | 123.5495 |
| kijk | k.Ei.k | look | 1049.7738 | 18 | 21.5365 |

Hard Word List:

| Word | PhoWord | Translation | Freq_per_million | PTAN | PTAF |
| --- | --- | --- | --- | --- | --- |
| vaas | v.2.s | vase | 4.5736 | 26 | 242.7665 |
| vaat | v.2.t | vascular | 1.4178 | 23 | 215.003 |
| been | b.eI.n | leg | 53.6252 | 18 | 566.7174 |
| dier | d.i.r | animal | 28.1046 | 25 | 657.8655 |
| haan | h.2.n | cock | 4.2763 | 18 | 647.2289 |
| koor | k.oU.r | choir | 6.8832 | 18 | 490.7083 |
| thee | t.eI | tea | 58.7934 | 19 | 742.9118 |
| peer | p.I.r | pear | 1.9895 | 21 | 216.9064 |
| poen | p.u.n | moolah | 11.7084 | 15 | 273.5062 |
| haai | h.2.j | shark | 9.4444 | 15 | 234.9067 |
| teen | t.eI.n | toe | 7.3863 | 20 | 305.4511 |
| nier | n.i.r | kidney | 3.9104 | 19 | 1461.1456 |
| maan | m.2.n | moon | 42.0998 | 21 | 1048.6892 |
| hoed | h.u.t | hat | 35.9483 | 31 | 1278.9684 |
| hiel | h.i.l | heel | 0.8461 | 17 | 373.9251 |
| boor | b.oU.r | drill | 3.4531 | 24 | 384.2521 |
| tong | t.O.N | tongue | 31.9007 | 16 | 188.3643 |
| wijn | 0.Ei.n | wine | 60.4399 | 25 | 563.5474 |
| touw | t.Vu | rope | 26.2523 | 27 | 618.4513 |
| cent | s.E.n.t | cent | 36.9317 | 17 | 195.3484 |
| halt | h.a.l.t | stop | 17.4939 | 23 | 168.271 |
| veer | v.I.r | spring | 3.4302 | 19 | 239.2102 |
| teer | t.I.r | tar | 3.3387 | 20 | 227.802 |
| zaal | z.2.l | room | 15.413 | 19 | 153.8191 |
| hout | h.Vu.t | wood | 23.5768 | 22 | 1313.7525 |
| maat | m.2.t | size | 69.1754 | 38 | 666.6011 |
| doel | d.u.l | goal | 77.4536 | 18 | 333.4038 |
| kier | k.i.r | crack | 0.9833 | 20 | 245.8985 |
| vouw | v.Vu | fold | 1.7151 | 24 | 375.8025 |
| maart | m.2.r.t | March | 8.3925 | 22 | 437.2696 |
| moer | m.u.r | nut | 9.2158 | 17 | 742.6229 |
| zaag | z.2.x | saw | 3.5445 | 15 | 175.9578 |
| goud | G.Vu.t | gold | 61.9949 | 22 | 338.0655 |
| mouw | m.Vu | sleeve | 4.1848 | 29 | 678.966 |
| tent | t.E.n.t | tent | 40.9335 | 20 | 171.8877 |
| wand | 0.a.n.t | wall | 3.293 | 28 | 463.0227 |
| mand | m.a.n.t | basket | 4.2992 | 25 | 173.9772 |
| moes | m.u.s | puree | 2.4011 | 17 | 284.0879 |
| haas | h.2.s | hare | 2.1725 | 33 | 317.3344 |
| wang | 0.a.N | cheek | 7.8894 | 16 | 765.4 |
| zool | z.oU.l | sole | 0.9147 | 15 | 434.8941 |
| bijl | b.Ei.l | ax | 9.2615 | 17 | 175.955 |
| gier | G.i.r | vulture | 1.0062 | 16 | 305.911 |
| meel | m.eI.l | flour | 1.6922 | 17 | 233.6884 |
| vacht | v.a.x.t | coat | 3.3158 | 19 | 157.3781 |
| moed | m.u.t | courage | 41.0479 | 23 | 522.0363 |
| noot | n.oU.t | note | 3.8189 | 18 | 1224.6128 |
| woud | 0.Vu.t | forest | 5.191 | 28 | 603.7991 |
| lach | l.a.x | laugh | 46.7191 | 20 | 173.158 |
| zuil | z.9y.l | column | 0.5488 | 15 | 158.6194 |
| zeil | z.Ei.l | sail | 6.9061 | 19 | 650.8264 |
| boon | b.oU.n | bean | 1.4178 | 19 | 341.0687 |
| heil | h.Ei.l | salvation | 5.717 | 20 | 520.0961 |
| pauw | p.Vu | peacock | 0.9605 | 25 | 334.1814 |
| dauw | d.Vu | dew | 0.9833 | 28 | 1482.717 |
| graad | G.r.2.t | degree | 4.0248 | 15 | 234.649 |
| laan | l.2.n | avenue | 1.2349 | 17 | 528.5336 |
| maag | m.2.x | stomach | 23.5539 | 17 | 610.9388 |
| doos | d.oU.s | box | 38.2809 | 23 | 188.8352 |
| mijl | m.Ei.l | mile | 15.3215 | 18 | 397.2535 |
